# The natural course of giant paraesophageal hernia and long-term outcomes following conservative management

**DOI:** 10.1177/2050640620953754

**Published:** 2020-08-24

**Authors:** Renske A B Oude Nijhuis, Margot van der Hoek, Jeroen M Schuitenmaker, Marlies P Schijven, Werner A Draaisma, Andreas J P M Smout, Albert J Bredenoord

**Affiliations:** 1 Amsterdam UMC, University of Amsterdam, Gastroenterology Endocrinology Metabolism, Meibergdreef 9, Amsterdam, the Netherlands; 2 Amsterdam UMC, University of Amsterdam, Department of Surgery, Meibergdreef 9, Amsterdam, the Netherlands; 3 Jeroen Bosch Hospital, Department of Surgery, den Bosch, The Netherlands

**Keywords:** Paraesophageal hernia, intrathoracic stomach, hiatal hernia, complication, acute symptoms, watchful waiting, conservative therapy

## Abstract

**Background:**

Accurate information on the natural course of giant paraesophageal hernia is scarce, challenging therapeutic decisions whether or not to operate.

**Objective:**

We aimed to investigate the long-term outcomes, including hernia-related deaths and complications (e.g. volvulus, gastrointestinal bleeding, strangulation) of patients with giant paraesophageal hernia that were conservatively managed, and to determine factors associated with clinical outcome.

**Methods:**

We retrospectively analysed charts of patients diagnosed with giant paraesophageal hernia between January 1990 and August 2019, collected from a university hospital in The Netherlands. Included patients were subdivided into three groups based on primary therapeutic decision at diagnosis. Radiological, clinical and surgical characteristics, along with long-term outcomes at most recent follow-up, were collected.

**Results:**

We included 293 patients (91 men, mean age 70.3 ± 12.4 years) with a mean duration of follow-up of 64.0 ± 58.8 months. Of the 186 patients that were conservatively treated, a total hernia-related mortality of 1.6% was observed. Hernia-related complications, varying from uncomplicated volvulus to strangulation, occurred in 8.1% of patients. Only 1.1% of patients included in this study required emergency surgery. Logistic regression analysis revealed the presence of symptoms (odds ratio (OR) 4.4, 95% confidence interval (CI) 1.8–20.6), in particular obstructive symptoms (vomiting, OR 15.7, 95% CI 4.6–53.6; epigastric pain, OR 4.4, 95% CI 1.2–15.8 and chest pain, OR 6.1, 95% CI 1.8–20.6) to be associated with the occurrence of hernia-related complications.

**Conclusions:**

Hernia-related death and morbidity is low in conservatively managed patients. The presence of obstructive symptoms was found to be associated with the occurrence of complications during follow-up. Conservative therapy is an appropriate therapeutic strategy for asymptomatic patients.

## Key summary

Summarise the established knowledge on this subject

- Information on the natural course of giant paraesophageal hernia is scarce. As a result, management and indication for elective surgical repair remains a topic of discussion.

What are the significant and/or new findings of this study?

- Hernia-related death and morbidity is low in conservatively treated patients.
- The presence of obstructive symptoms was found to be associated with the occurrence of complications during follow-up.
- Conservative therapy is an appropriate therapeutic strategy for asymptomatic patients.

## Introduction

Diaphragmatic herniation is a common condition involving the gastrointestinal tract. It is characterised by a protrusion of the stomach and/or other intra-abdominal content into the chest cavity through a widening between both slings of the right crus of the diaphragm.^1^ Hiatal hernia can be categorised in four anatomical patterns.^2^ By far the most common type of hiatal hernia and strongly associated with gastroesophageal reflux is a sliding or type I hiatal hernia in which the gastroesophageal junction migrates above the diaphragm.^1^ Type II or a paraesophageal hernia represents only 5% of all hiatal hernias, with herniation of the gastric fundus adjacent to a normally positioned esophagogastric junction. Type III hernia is a combination of both types I and II. Often, due to a progressive enlargement of hiatus and herniation, these hernias tend to be of considerable size, taking up a great part of the thoracic cavity.^3^ Type IV represents a more complex type of hernia, with complete migration of other intra-abdominal viscera such as small bowel or colon in the hernia sac. Definitions of the terms ‘intrathoracic stomach’ or ‘giant’ paraesophageal hernia appear inconsistently in the literature, but most authors limit these terms to those paraesophageal hernias having greater than one-third of the stomach in the thorax.^1,3–6^

A giant paraesophageal hernia can present itself in a wide variety of forms, ranging from an incidentally detected hernia without symptoms, to a gastric volvulus with risk of ischaemia. Dysphagia, reflux or obstructive symptoms such as postprandial pain and vomiting are reported.^1^ In addition, respiratory symptoms as a result of pulmonary compression, or gastrointestinal bleeding due to reflux esophagitis and ulceration may occur. A gastric volvulus is a very rare but major complication associated with paraesophageal hernia, and may lead to gastric bleeding, incarceration and strangulation causing bowel obstruction, ischaemia and/or perforation.^7,8^ The need for surgical correction in asymptomatic, or mildly symptomatic patients is an ongoing matter of debate. Despite the fact that the finding of giant paraesophageal hernia is incidental in a large subset of patients, it is believed that potentially life-threatening complications may occur if the hernia is not surgically managed.^9^ However, the majority of this patient population is often of advanced age with extensive comorbidity, making them poor surgical candidates.

Traditionally, elective surgery was often advocated for every patient, in spite of symptoms, with the objective of preventing acute complications and to avoid significant mortality and morbidity associated with emergency surgery.^7,8,10–14^ While more recent series suggest that the occurrence of life-threatening complications in untreated patients as well as the mortality rates for emergency surgery are much lower than initially estimated.^15–18^ However, all current knowledge on the true natural course of a giant paraesophageal hernia derives from older, small series with a limited duration of follow-up. Due to the paucity of long-term observational cohort studies, information on the natural course and complication risk of untreated giant paraesophageal hernia is scarce and the indication for elective hernia repair in mildly symptomatic patients remains a subject of discussion. In the present study we were able to identify a substantial cohort of conservatively treated patients with giant paraesophageal hernia over almost three decades. Our aim was to describe the long-term outcomes of these patients and to determine characteristics associated with clinical outcome.

## Methods

### Study design

We retrospectively studied a cohort of patients diagnosed and followed up at the gastroenterology and surgery departments of the Amsterdam University Medical Center. Patients diagnosed with a giant paraesophageal hernia were identified through radiology reports. Electronic charts were critically assessed and relevant data were extracted. Missing chart documentation at follow-up was obtained by means of telephone interviews.

### Patient selection

All radiography, computed tomography (CT) and barium oesophagogram reports between January 1990 and August 2019 were searched with a query based on the keywords ‘intrathoracic stomach’ and ‘paraesophageal hernia’. The full search query is detailed in Supplementary Table 1. Electronic charts of the retrieved patient numbers were independently screened for eligibility by two reviewers (RON and MH). In case of uncertainty, charts were re-reviewed by a third reviewer (AJB) until consensus was reached. We included adult patients with the radiological diagnosis of a giant paraesophageal hernia, defined as herniation of at least one-third of the stomach into the thoracic cavity.^1,3–6^ Exclusion criteria were: the presence of congenital or traumatic hernia or a history of oesophageal surgery or radiation therapy. Relevant data from eligible patients were extracted and registered in an electronic patient record system (Castor EDC, The Netherlands). Extracted information included demographics (e.g. age, sex, body mass index (BMI)), clinical characteristics (age at symptom onset, age at diagnosis, medical history, medication use and intoxications), and disease-specific characteristics (symptoms, radiological and endoscopic findings).

### Clinical and radiological characteristics

Symptoms were extracted from patient charts and scored as either present or absent, based on the clinical assessment and recording of the treating physician at the time of diagnosis and at latest follow-up. Extracted symptoms included: epigastric pain, heartburn, dysphagia, chest pain, weight loss, bloating, dyspepsia, postprandial fullness, regurgitation, dyspnoea, haematemesis and belching. Both age at diagnosis and age at onset of symptoms were retrieved. Endoscopic data were extracted from endoscopy reports. Reports were screened for signs of reflux oesophagitis, Barrett’s oesophagus, the presence of Cameron lesions, malignancies and ulcer disease. Radiology reports were screened for hernia size, hernia type (sliding, paraesophageal or combined) and the involvement of other abdominal organs as reported by the radiologist.

### Treatment characteristics

Included patients were subdivided into three groups based on the primary therapy they received; elective surgery, emergency surgery or conservative therapy. Conservative treatment was defined as any type of medical treatment other than surgery. In the case of primary surgical treatment, procedure time, surgical approach (abdominal or thoracic), type (laparotomy or laparoscopic), addition of anti-reflux procedure, American Society of Anesthesiologists (ASA) physical status classification were extracted. The decision to operate in the elective setting was made by the treating physician for each patient individually and based on the type and extent of symptoms, a patient’s quality of life and surgical risk.

### Long-term outcomes

As the main objective of this study was to explore the natural history of giant paraesophageal hernia, we extracted follow-up data for the conservatively managed patients. Data on the presence and type of symptoms, current medication use, occurrence of any hernia-related events or complications during the course of follow-up were collected at the time of latest available follow-up visit. All hernia-related events that required acute intervention or hospital admission were reported as a complication and were divided into: obstructive complications with or without ischaemia, oesophageal or gastric perforation, cardiac or respiratory failure and acute bleeding. Finally, the vital status and cause of death were extracted. In deceased patients, in whom the cause of death could not be obtained, general practitioners were contacted for information. In the case of missing follow-up documentation, patients were contacted and questionnaires by telephone were conducted to assess current health status, the presence of symptoms, the occurrence of any (acute) hernia-related events, or hospital admissions. An uneventful follow-up was defined as the absence of complications, elective surgical hernia repair, symptom progression or hernia-related death at the end of follow-up.

### Ethics

The study protocol was reviewed by the local institutional review board (IRB) and as this was a retrospective study and patients were not exposed to any additional interventions for the study purpose, it was confirmed that the Medical Research Involving Human Subjects Act did not apply (reference number W19_228#19.274).

### Statistical analysis

SPSS statistics (version 24; SPSS, Chicago, IL, USA) was used for statistical analysis. Descriptive statistics were presented as a percentage for categorical data and as means with standard deviations for continuous variables. Due to retrospective non-standardised data collection, not all included patients had a complete dataset, therefore all results are presented as percentages of the total number of patients for whom the concerning variable was available. Mann–Whitney U or χ^2^ tests were used to compare variables when appropriate. Annualised risk rates were expressed as percentages and calculated by the number of hernia-related events divided by the number of patient-years follow-up. Of note, these annual rates were calculated under the assumption that annual risk is constant over time and independent of disease duration. To explore factors associated with the occurrence of hernia-related complications univariate logistic regression analysis was performed.

## Results

### Patient selection

**Figure 1.**
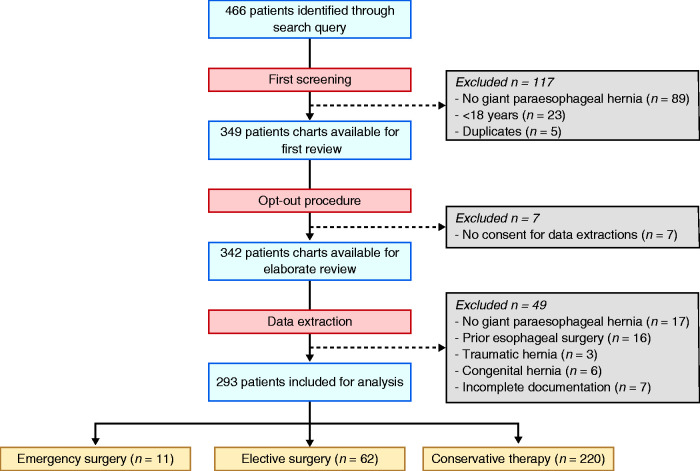
Flow chart of case findings.

We retrieved a cohort of 466 patients with a potential radiological diagnosis of giant paraesophageal hernia. After an initial screening and the removal of duplicates, 342 patients with a confirmed diagnosis of giant paraesophageal hernia were identified. Patients younger than 18 years at the time of diagnosis (*n* = 23) and patients who did not give consent for data extraction (*n* = 7) were excluded. After critical appraisal of these 342 patient files, another 49 patients were excluded because of congenital (*n* = 6) or traumatic hernia (*n* = 3), less than one-third of the stomach in the chest cavity (*n* = 17), a history of oesophageal surgery (*n* = 16), or oesophageal radiation therapy (*n* = 3). Seven patients were excluded due to incomplete or missing chart documentation. Ultimately, 293 patients met the diagnostic definition of a giant paraesophageal hernia and fulfilled our inclusion and exclusion criteria. Subject identification and recruitment is presented in Figure 1.

### Patient characteristics

Of the 293 included patients 91 (31.1%) were men. Patients’ mean age at diagnosis was 70.3 ± 12.4 years. Of the 289 patients for whom the medical history was known, a subset had chronic comorbidities, including ischaemic heart disease (*n* = 40, 13.8%), arterial vascular disease (*n* = 34, 11.8%), chronic obstructive pulmonary disease (*n* = 32, 11.1%) or a history of diabetes mellitus (*n* = 30, 10.4%). A complete overview of patients’ characteristics and medical history is presented in Table 1.

**Table 1.** Baseline characteristics in patients with giant paraesophageal hernia.

	Total study population (*n* = 293)			Conservative(*n* = 220)	Surgical(*n* = 62)	*P* value
	*n*^a^/*N*^b^	%	Mean(SD) or median (IQR)	*n*^a^/*N*^b^ (%)	*n*^a^*/N*^b^ (%)	
Sex						0.867
Male	91/293	31.1		65/220 (29.5)	19/62 (30.6)	
Female	202/293	68.9		155/220 (70.5)	43/62 (69.4)	
Age at diagnosis (years), mean (SD)			70.3 (12.4)	73.0 (11.6)	61.8 (9.6)	<0.001
Caucasian	220/293	75.1		165/220 (75.0)	47/62 (75.8)	0.897
BMI,^c^ median (IQR)			27.02 (4.7–31.1)	28.9 (24.7–31.1)	27.7 (25.0–31.6)	0.360
ASA ≥3	86/288	29.9		79/215 (36.7)	2/62 (3.2)	<0.001
Intoxications						
History of smoking	76/211	36.0		56/164 (34.1)	18/62 (40.0)	0.467
Alcohol use >2 units per day	21/196	10.7		18/152 (11.8)	3/43 (7.0)	0.364
Medical history						
Cardiac disease	40/289	13.8		34/216 (15.7)	4/62 (6.5)	0.061
Vascular disease	34/289	11.8		27/216 (12.5)	5/62 (8.1)	0.335
COPD	32/289	11.1		24/216 (11.1)	8/62 (12.9)	0.697
Diabetes mellitus	30/289	10.4		23/216 (10.6)	7/62 (11.3)	0.886
Concomitant oesophageal carcinoma	9/293	3.1		8/220 (3.6)	1/62 (1.6)	0.423

ASA: American Society of Anesthesiologists; BMI: body mass index; IQR: interquartile range; COPD: chronic obstructive pulmonary disease; SD: standard deviation.

^a^Number of patients.

^b^Total number of patients in whom data could be obtained.

^c^*n* = 173.

### Symptoms and endoscopic findings

The majority of patients (*n* = 179, 61.1%) presented with symptoms at diagnosis. Heartburn (*n* = 61, 21.5%), respiratory symptoms (*n* = 61, 21.5%), epigastric pain (*n* = 51, 18.0%) and dysphagia (*n* = 42, 14.8%) were the most frequently reported symptoms (Table 2). Other less commonly exhibited symptoms were nausea or vomiting (*n* = 39, 13.7%), chest pain (*n* = 38, 13.4%), weight loss (*n* = 24, 8.5%), regurgitation (*n* = 22, 7.7%), or postprandial fullness (*n* = 15, 5.3%). Twenty-five (8.5%) patients presented with one or multiple hernia-related complications at the time of diagnosis. Obstruction and gastrointestinal bleeding were predominantly reported (60.0% and 24.0%, respectively). A subset of patients (38.9%) presented asymptomatically. In 166 (59.2%) patients the finding of a giant paraesophageal hernia was discovered incidentally. Iron deficiency anaemia was found in 50 of the 274 patients (18.2%) in whom laboratory results were reported. Upper endoscopy was performed in 111 patients. We identified 16 patients (14.4%) with reflux oesophagitis, 13 patients (11.7%) with concomitant Barrett’s oesophagus, seven patients (6.3%) with Cameron lesions and four patients (3.7%) with gastric ulcers at endoscopic inspection.

**Table 2.** Clinical, endoscopic and radiological characteristics of patients with giant paraesophageal hernia.

	Total study population (*n* = 293)		Conservative(*n* = 220)	Surgical(*n* = 62)	*P* value
	*n*^a^/*N*^b^	%	*n*^a^/*N*^b^ (%)	*n*^a^*/N*^b^ (%)	
Symptoms at diagnosis	179/293	61.1			
Asymptomatic	114/293	38.9	114/220 (51.8)	0/62(0.0)	<0.001
Incidental finding	166/280	59.3	159/215(74.0)	4/55(7.3)	<0.001
Type of symptoms					
Heartburn	61/284	21.5	36/213 (16.9)	25/61 (41.0)	<0.001
Respiratory symptoms	61/284	21.5	41/213 (19.2)	18/61 (29.5)	0.086
Epigastric pain	51/284	18.0	21/213 (9.9)	26/61 (42.6)	<0.001
Dysphagia	42/284	14.8	17/213 (8.0)	24/61 (39.3)	<0.001
Nausea and/or vomiting	39/284	13.7	21/213 (9.9)	13/61 (21.3)	0.017
Chest pain	38/284	13.4	20/213 (9.4)	16/61 (26.2)	0.001
Weight loss	24/284	8.5	9/213 (4.2)	14/61 (23.0)	<0.001
Regurgitation	22/284	7.7	7/213 (3.3)	15/61 (24.6)	<0.001
Postprandial fullness	15/284	5.3	8/213 (3.8)	7/61 (11.5)	0.019
Belching	6/284	2.1	3/213 (1.4)	3/61 (4.9)	0.099
Hernia-related complications	25^c^/293	8.5	11/220 (5.0)	3/62 (4.8)	0.959
Obstruction	15	60.0			
Gastrointestinal bleeding	6	24.0			
Obstruction with ischaemia	4	16.0			
Respiratory/cardiac compression	2	8.0			
Gastric/oesophageal perforation	1	4.0			
Laboratory findings					
Iron deficiency anaemia	50/274	18.2	42/208 (20.2)	7/57 (12.3)	0.173
Endoscopic findings					
Reflux oesophagitis	16/111	14.4	7/68 (10.3)	8/40 (20.0)	0.159
Cameron lesions	7/111	6.3	3/68 (4.4)	4/40 (10.0)	0.255
Barrett’s oesophagus	13/111	11.7	7/68 (10.3)	5/40 (12.5)	0.725
Gastrointestinal ulcer(s)	4/111	3.6	4/68 (5.9)	0/40 (0.0)	0.118

^a^Number of patients.

^b^Total number of patients in whom data were obtained.

^c^Number of patients with one or multiple hernia-related complications at diagnosis.

### Radiological characteristics

Diagnosis was established with CT in 52 (17.7%) patients (Table 3). Fifty-six (19.1%) patients and 91 (32.1%) patients were diagnosed by means of barium oesophagram and chest radiography, respectively. In the majority of patients (*n* = 94, 32.1%) a combination of diagnostic tests (e.g. CT, oesophagram and radiography) were performed to establish the diagnosis of giant paraesophageal hernia. Type III hiatal hernia was most often reported (90.8%). Type IV was described in only 27 (9.2%) patients.

**Table 3.** Radiological diagnosis of patients with giant paraesophageal hernia.

	Total study population(*n* = 293)	Conservative(*n* = 220)	Surgical(*n* = 62)	*P* value
	*n*^a^/*N*^b^ (%)	*n*^a^/*N*^b^ (%)	*n*^a^*/N*^b^ (%)	
Radiological diagnosis				
CT scan	52/293 (17.7)			
Chest radiograph	91/293 (31.1)			
Barium oesophagram	56/293 (19.1)			
Combination of tests listed above	94/293 (32.1)			
Hernia anatomy				0.976
Type III hiatal hernia	266/293 (90.8)	202/220 (91.8)	57/62 (91.9)	
Type IV hiatal hernia	27/293 (9.2)	18/220 (8.2)	5/62 (8.1)	

CT: computed tomography.

^a^Number of patients.

^b^Total number of patients in whom data were obtained.

### Primary therapy

All included patients were categorised based on the primary therapeutic decision at the time of diagnosis. The characteristics of patients who received conservative treatment (*n* = 220) and elective surgery (*n* = 62) are displayed separately in Tables 1, 2 and 3. The characteristics of patients who underwent emergency surgery at baseline (*n* = 11) are displayed in Supplementary Table 2. In patients who were conservatively treated, the majority of patients (*n* = 129, 58.6%) used or were started on pharmacological therapy. Proton pump inhibitors were most frequently used (54.5%), followed by H_2_-receptor antagonists (6.4%) and prokinetic drugs (5.0%). Twenty-five (8.5%) patients presented with hernia-related complications at the time of diagnosis, of whom 11 (3.8%) (median age 72 years, interquartile range (IQR) 46–74) underwent emergency surgery. These complications are specified in Supplementary Table 3. One patient underwent a laparotomic partial gastric resection. In the remaining 10 patients an emergency hernia correction was performed, of whom eight underwent an open procedure. In the elective surgery group, specific information on the type of surgical procedure was available in 58 patients. The majority of patients (70.6%) underwent laparoscopic hernia repair. An anti-reflux procedure was performed in 42 out of 58 patients (72.4%), this was a Toupet fundoplication in half of the cases. Cruroplasty was performed in all 58 patients, while mesh-based reinforcement was used in only 8.6% of patients. The surgical characteristics of patients treated electively or emergently are displayed in Supplementary Table 4.

### Differences in surgically and conservatively treated patients

Conservatively treated patients were younger (*P* < 0.001) and had higher ASA scores (≥3) (*P* < 0.001) (Table 1). With regard to symptoms, patients who underwent elective surgery were symptomatic in all cases, whereas 48.2% of patients in the conservative treatment group presented with symptoms (*P* < 0.001) (Table 2). The majority of symptoms; for example, dysphagia, heartburn, epigastric pain, regurgitation, postprandial fullness, chest pain and nausea were predominantly observed in patients who were treated with an elective operation.

### Clinical course and long-term follow-up in the elective surgery group

In the elective surgery group, intraoperative or postoperative complications occurred in 12 (22.2%) and nine (16.7%) patients, respectively (Supplementary Table 5). Follow-up data could be obtained for 60 of the 62 patients who underwent elective surgery. The median follow-up time in this group was 33 (IQR 12–106) months. After surgery, 33 (53.3%) patients became symptomatic, this included any recurrent or new postoperative complaints during the postoperative course. Of these patients, hernia recurrence was confirmed by radiology in 19 (31.7%) patients, of whom 11 patients underwent redo surgery. Two patients presented with acute symptoms and underwent emergency surgery; both patients presented with gastric perforation due to gastric obstruction with ischaemia. There were no (hernia-related) deaths in the elective surgery group.

### Long-term follow-up in conservatively treated patients

**Figure 2.**
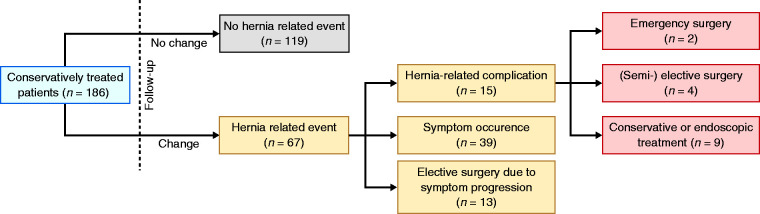
Long-term outcomes in the 186 conservatively treated patients in whom follow-up data could be obtained.

Follow-up data could be obtained in 186 conservatively treated patients and are summarised in Figure 2. The mean duration of follow-up of this group was 58 (IQR 31–106) months. The majority of patients (64.0%) reported no changes in clinical course or any hernia-related events. Sixty-seven (36.0%) patients experienced a hernia-related event in the course of follow-up, of whom 39 (58.2%) patients reported symptom progression that could still be managed conservatively. In 13 (7.0%) patients symptoms worsened in such a way that elective hernia repair was indicated. Hernia-related complications occurred in 15 (8.1%) patients, of which three (1.6%) were classified as gangrenous complications (Supplementary Table 6). Two (1.1%) patients underwent emergency surgery because of strangulation and gastric perforation. The corresponding annual risks for requiring emergency surgery and developing a hernia-related complication were 0.2% per annum and 1.7% per annum, respectively. One of the patients died shortly after surgery due to septic shock. Two patients did not undergo emergency surgery because of extensive comorbidity and died from their complications; one patient from obstruction with respiratory failure and the other due to severe gastric bleeding. The remaining 11 patients could be managed either semi-electively (*n* = 4) or conservatively (*n* = 7). Of all 220 conservatively treated patients, 96 (43.6%) patients had died during the course of follow-up. We were able to obtain the cause of death in the majority (83.3%, *n* = 80) of these patients. As mentioned earlier, three (1.6%) patients of the 186 patients in whom follow-up data could be obtained, eventually died from a hernia-related complication.

### Risk factors for hernia-related complications in conservatively treated patients

To determine risk factors for hernia-related complications in patients who were conservatively managed, we performed a logistic regression analysis with the occurrence of complications as a dependent variable (Table 4). Univariate analysis identified the presence of symptoms at diagnosis (OR 4.44; 95% CI 1.21–16.31; *P* = 0.025), epigastric pain (OR 4.37; 95% CI 1.21–15.76; *P* = 0.024), chest pain (OR 6.07; 95% CI 1.79–20.62; *P* = 0.004), vomiting (OR 15.70; 95% CI 4.60–53.56; *P* < 0.001) and Cameron lesions (OR 17.00; 95% CI 1.33–216.67; *P* = 0.029) as risk factors for the occurrence of hernia-related complications.

**Table 4.** Logistic regression analysis for identifying risk factors for ‘hernia-related complications’ in conservatively treated patients.

Demographic and clinical characteristics	Univariable		
	OR	95% CI	*P* value
Male sex	2.117	0.729–6.125	0.168
Age	1.019	0.969–1.071	0.462
BMI	0.991	0.912–1.076	0.830
ASA ≥3	0.273	0.059–1.261	0.096
Smoking	1.533	0.502–4.658	0.453
Alcohol use >2 units/day	2.850	0.688–11.799	0.149
Use of risk medication^a^	0.331	0.041–2.643	0.297
Disease-specific characteristics			
Hernia type IV	2.477	00.490–12.515	0.272
Complete herniation of stomach in chest cavity	2.183	0.733–6.507	0.161
Presence of symptoms at diagnosis	4.444	1.211–16.312	0.025
Duration of symptoms	2.183	0.733–6.507	0.161
Type of symptoms at diagnosis			
Dysphagia	2.153	0.431–10.749	0.350
Postprandial fullness	4.472	0.813–24.588	0.085
Heartburn	1.151	0.303–4.366	0.837
Respiratory symptoms	1.107	0.292–4.197	0.085
Regurgitation	–	–	–
Chest pain	6.071	1.788–20.617	0.004
Epigastric pain	4.371	1.213–15.757	0.024
Belching	12.769	0.755–216.100	0.078
Weight loss	1.758	0.201–15.402	0.610
Nausea/vomiting	15.700	4.602–53.566	<0.001
Iron deficiency anaemia	0.593	0.127–2.770	0.506
Endoscopic findings			
Reflux oesophagitis	1.714	0.167–17.626	0.650
Cameron lesions	17.000	1.334–216.666	0.029
Barrett	–	–	–
Ulcer(s)	3.571	0.285–44.718	0.324

ASA: American Society of Anesthesiologists; BMI: body mass index; CI: confidence interval; OR: odds ratio.

^a^Risk medication was defined as medication associated with a potentially damaging effect on gastric mucosa, such as anticoagulants, corticosteroids, selective serotonin re-uptake inhibitors or non-steroidal anti-inflammatory drugs.

## Discussion

The management and indication for surgical repair of giant paraesophageal hernias remained a topic of discussion for decades. Despite ongoing controversies, accurate information on the natural course of paraesophageal hernia is scarce. In the present study we were able to identify a large cohort of patients over almost three decades. A comprehensive analysis of 293 patients was conducted and, radiological, clinical, endoscopic and surgical features were identified and stratified by primary therapeutic decision. The results of this study strongly support the view that elective repair of a giant paraesophageal hernias is not required in all patients. We demonstrated that hernia-related death in conservatively treated patients, followed up for a median of 58 months, is rare; in 186 patients, a total hernia-related mortality of 1.6% was observed. Although hernia complications, varying from uncomplicated volvulus to strangulation, occurred in 8.1% of our patients, only 1.1% of these patients required emergency surgery. The majority could be managed either endoscopically or conservatively. We demonstrated that symptomatic patients have a 4.4-fold higher risk of developing a hernia-related complication. In particular, obstructive symptoms, including epigastric pain and vomiting, were found to be associated with the occurrence of complications at a later time. In addition, as a result of the generally high age in this patient group, almost all of the deceased patients in our cohort eventually died from other comorbid diseases.

The dictum that all paraesophageal hernias should be repaired electively irrespective of symptoms, derived from early reports that raised concerns of high complication rates, suffered from patients left untreated.^7,8,19^ The occurrence of potentially life-threatening complications were described in up to 29% of the patients.^7,8^ However, in the years that followed, several surgeons and investigators have been questioning the benefit of performing elective hernia repair in mildly symptomatic or asymptomatic patients. Allen and colleagues described 23 unoperated patients, who were followed for a mean of 6.5 years. None developed hernia-related complications or required emergency surgery.^15^ Treacy and Jamieson evaluated 29 untreated patients, and inspite of 13 (45%) patients who required elective surgery for progression of symptoms, none had to be treated emergently.^16^ More than a decade later, the surgical viewpoint was further undermined by a report using population-based decision analysis modeling to conclude that the mortality rate of elective hernia repair was 1.4%, whereas the annual probability of developing a hernia-related complication was only 1.1%.^17^ A more recent study showed that gangrenous complications occurred in only 0.9% of patients admitted from 1998 to 2008 for giant paraesophageal hernia.^20^ This is in line with our findings; of the unoperated patients, only 1.6% developed volvulus with strangulation or ischaemia. Of note, we found a higher total complication rate of 8% for untreated paraesophageal hernia than Stylopoulus and colleagues, as they specified complications only as obstructed or strangulated hernia, whereas we also included bleeding from reflux oesophagitis or gastrointestinal ulcers.^17^ Nevertheless, our estimated rate of 1.1% for requiring emergency surgery is in accordance with the results from the aforementioned study. In this respect, our findings are in keeping with the more recent reports that suggest that symptom progression is slow and is less likely to evolve to acute symptoms than previously expected.

As mentioned earlier, the rationale behind the shifting surgical dictum is twofold; besides the low complication rates in unoperated patients, the more recent studies also demonstrated that mortality for emergency surgery was much lower than initially believed. Previously, early studies advocated elective surgery in all patients because of reported mortality rates up to 17% for emergency surgery,^8^ whereas the more recent studies have shown that the mortality of emergency surgical repair was presumably overestimated in early reports, and is more likely to be between 0.4% and 5%.^17,21^ In line with this, we found rather high complication rates in our elective surgery group, most likely explained by the fact that we included a subset of patients who underwent surgery in the early 1990s, while more recent series show that outcomes after elective surgery have improved tremendously with new advancements in laparoscopic or robot-assisted hernia repair.^22^ Our study shows that the overall risk of the occurrence of acute complications of giant paraesophageal hernia in conservatively managed patients in time is low. Therefore, we support the standpoint that conservative management is an appropriate strategy for asymptomatic or moderately symptomatic patients with giant paraesophageal hernia. This applies in particular for elderly or frail patients, in whom this condition is most commonly found and who often have extensive comorbidities. A large subset of our conservatively managed patients died of other comorbid diseases before the end of follow-up. Hence, besides the fact that these patients are often poor surgical candidates to begin with, another argument for deferring elective surgery in this group is that the vast majority will most likely die from other comorbid diseases.

Many considerations must be taken into account when formulating therapeutic strategies for patients with giant paraesophageal hernia, and it is with good reason that hernia repair of this subgroup remains one of the most widely debated and controversial areas in surgery. What recommendations can be made in terms of therapeutic decision-making? First, standard elective operation is not necessarily required in all mild to moderately symptomatic patients. Especially in older patients, who are in general considered to be less fit for surgery, watchful waiting is a valuable therapeutic alternative. Pharmacological or endoscopic therapy may be sufficient for symptom control in a subset of patients. Second, symptomatic patients should be consulted by a foregut surgeon to discuss definitive surgical repair. The decision to operate in the elective setting should largely depend on the type and extent of a patient’s symptoms. Symptoms secondary to mechanical obstruction are more concerning for subsequent volvulus, whereas non-obstructive symptoms including gastroesophageal reflux can often be managed pharmacologically. We emphasise the importance of a thorough clinical evaluation and counseling by an upper gastrointestinal surgeon, in which the risk–benefit profile of definitive repair versus observation is weighed, taking into account the extent and type of symptoms, hernia anatomy, a patient’s age and perioperative risk.

This study has some limitations. First, the findings of this study should be appraised while keeping in mind that patients were selected from one academic healthcare centre, which could have led to selection bias. Second, the results are based on retrospective analysis of patients’ charts in which data were not uniformly registered. Therefore, we were unable to obtain complete and standardised datasets of all patients. In addition, we had to rely on the clinical evaluation, registration and decision of the treating physicians, which may have induced bias as well. In line with this, the number of symptomatic patients may be underestimated. Expert opinion suggests that truly asymptomatic paraesophageal hiatal hernias do exist, but are rare. Nevertheless, to minimise these limitations, stringent inclusion and exclusion criteria were used, charts were critically appraised by two or three reviewers, and missing chart documentation at follow-up was obtained through telephone interviews.

In conclusion, this is the largest available study reporting on the natural course of giant paraesophageal hernia. We showed that hernia-related death and morbidity is low in conservatively treated patients. Therefore, conservative management is an appropriate therapeutic strategy for asymptomatic patients.

## Supplemental Material

sj-pdf-1-ueg-10.1177_2050640620953754 - Supplemental material for The natural course of giant paraesophageal hernia and long-term outcomes following conservative management Click here for additional data file. Supplemental material, sj-pdf-1-ueg-10.1177_2050640620953754 for The natural course of giant paraesophageal hernia and long-term outcomes following conservative management by Renske AB Oude Nijhuis, Margot van der Hoek, Jeroen M Schuitenmaker, Marlies P Schijven, Werner A Draaisma, Andreas JPM Smout and Albert J Bredenoord in United European Gastroenterology Journal
