# Supplementary material for: The natural course of giant paraesophageal hernia and long-term outcomes following conservative management
Source: United European Gastroenterol J. 2020 Aug 24;8(10):1163–73. doi: 10.1177/2050640620953754 (PMC7724529; doi:10.1177/2050640620953754)
Supplement: sj-pdf-1-ueg-10.1177_2050640620953754 - Supplemental material for The natural course of giant paraesophageal hernia and long-term outcomes following conservative management [file sj-pdf-1-ueg-10.1177_2050640620953754.pdf]

SUPPLEMENTAL MATERIAL

Supplementary table 1. Query used to search all radiology reports from January 1990 until August 2019 with the keywords 'intrathoracic stomach' and 'paraesophageal hernia'.

1)
'Intrathoracale maag'
'intrathoracaal gelegen maag'

Simplified to:
%Intrathoraca%maag%

2)
'(grote*) paraoesofageale (hiatus*)hernia'
'para-oesofageale hernia'
'paraoesophageale hernia'
'para-oesophageale hernia'

Simplified to:
%para%oeso%ageale%hernia%

Supplementary table 2. Baseline characteristics of patients who underwent emergency surgery at baseline.

Demography	n[a]/N[b]	%	Mean(SD)
Male sex			
- male	7/11	63.6	
- female	4/11	36.4	
Age at diagnosis (years), mean (SD)			65.5 (18.9)
Caucasian	8/11	72.7	
BMI[c], median [IQR]			23.1 [3.3]
ASA ≥3	5/11	45.5	
Medical history			
Cardiac disease	2/11	18.1	
Vascular disease	2/11	18.1	
COPD	0/11	0.0	
Diabetes mellitus	0/11	0.0	
Concomitant esophageal carcinoma	0/11	0.0	
Symptoms at diagnosis	11/11	100.0	
Heartburn	0/11	0.0	
Respiratory symptoms	2/11	18.1	
Epigastric pain	4/11	36.4	
Dysphagia	1/11	9.1	
Nausea and/or vomiting	5/11	45.5	
Chest pain	2/11	18.1	
Weight loss	1/11	9.1	
Regurgitation	0/11	0.0	
Postprandial fullness	0/11	0.0	
Belching	0/11	0.0	
Iron deficiency anemia	1/9	11.1	
Endoscopic findings			
Reflux esophagitis	1/3	33.3	
Cameron lesions	0/3	0.0	
Barrett's esophagus	1/3	33.3	
Gastrointestinal ulcer(s)	0/3	0.0	
Radiologic diagnosis			
CT scan	5/11	45.5	
Chest radiograph	5/11	45.5	
Barium esophagram	1/11	9.1	
Hernia anatomy			
Type III hiatal hernia	7/11	63.6	
Type IV hiatal hernia	4/11	36.4	

a. Number of patients.
b. Total number of patients in whom data could be obtained.
c. n = 2
ASA, American Society of Anesthesiologists classification; BMI, body mass index; COPD, Chronic Obstructive Pulmonary Disease; IQR, Inter Quartile Range; SD, Standard Deviation.

Supplementary table 3. Patients with an acute complication that underwent emergency surgery at baseline

Patient	*Age (years)*	*Sex*	*Type of complication (n = 11)*
1	70	M	Obstruction
2	74	M	Obstruction with ischemia
3	74	F	Obstruction
4	70	M	Obstruction
5	28	M	Obstruction
6	85	M	Cardiac compression
7	88	F	Obstruction
8	46	F	Obstruction with ischemia
9	72	M	Gastrointestinal bleeding
10	40	M	Obstruction with ischemia and gastrointestinal bleeding
11	73	F	Obstruction with ischemia and perforation

Supplementary table 4. Surgical characteristics in patients who underwent elective and emergency surgery at baseline

	Elective surgery (n = 60)		Emergency surgery (n = 11)	
	n[a]/N[b]	%	n[a]/N[b]	%
Type of repair				
Laparoscopic hernia repair	41/58	70.6	2/11	18.2
Open hernia repair	17/58	29.3	8/11	72.7
Open gastric resection	-	-	1/11	9.1
Hiatal closure				
Suture-based	58/58	100.0	11/11	100.0
Mesh-reinforced	5/58	8.6	0/11	0.0
Anti-reflux procedure	42/58	72.4	3/11	27.3
Toupet	21	50.0	1	33.3
Nissen	15	35.7	2	66.7
Dor	1	2.4	0	0.0
Unknown	5	11.9	0	0.0
Surgical details				
Transabdominal approach	58/58	100.0	11/11	100.0
Gastropexy	36/49	73.5	6/11	54.5
Hernia sac excision	36/54	66.7	8/11	72.7
Operation time in minutes, mean (SD)	135.3 [c]	55.1	128.1	32.0

a. Number of patients.
b. Total number of patients of whom data was obtained
SD, standard deviation

Supplementary table 5. Perioperative outcomes in patients who underwent elective and emergency surgery at baseline

	Elective surgery	Emergency surgery

	(n = 60)		(n = 11)	
	n[a]/N[b]	%	n[a]/N[b]	%
Intraoperative complications	12/54	22.2	2/11	18.2
· Splenic laceration	4		1	
· Esophageal or gastric perforation	2		0	
· Opening pleura	3		1	
· Vagal nerve ligation	2		0	
· Subcutaneous emphysema	1		0	
Postoperative complications	9/54	16.7	2/11	18.2
· Cardiac arrhythmia	2		-	
· Wound infection	1		-	
· Bleeding	1		-	
· incisional hernia	4		-	
· Pneumonia	1		1	
· Infection of haematoma	-		1	
Length of hospital stay in days, median(IQR)*	5.0 [c]	[3.0-100]	9.0	[7.5-19]
In-hospital mortality	0/62	0	2/11	18.2
Sepsis/SIRS	-		2	

a. Number of patients.
b. Total number of patients of whom data was obtained.
c. in 46 patients in whom variable could be obtained
* $p = 0.01$
SIRS, systemic inflammatory response syndrome

Supplementary table 6. Occurrence of hernia-related complications and subsequent management in patients that received conservative therapy at baseline

Patient	*Complication (n=15)*	*Treatment*
1	Obstruction with respiratory failure	Deceased from complication in combination with extensive comorbidity
2	Recurrent episodes of strangulation	Endoscopic desufflation followed by elective surgery
3	Gastrointestinal bleeding	Elective surgery
4	Volvulus with ischemia	Endoscopic desufflation followed by elective surgery
5	Volvulus	Conservative
6	Gastrointestinal bleeding	Conservative
7	Gastrointestinal bleeding	Conservative
8	Gastrointestinal bleeding	Conservative
9	Gastrointestinal bleeding	Conservative
10	Strangulation with perforation	Emergency surgery, deceased after surgery due to septic shock
11	Gastric bleeding	Deceased from complication in combination with extensive comorbidity
12	Obstruction	Elective surgery
13	Gastric perforation due to gastric ulcer	Emergency surgery
14	Volvulus and respiratory failure	Endoscopic desufflation
15	Volvulus	Conservative
